# Copaiba Oil–Resin Reduces the Alveolar Bone Damage Triggered by Apical Periodontitis in Rats

**DOI:** 10.1111/iej.70063

**Published:** 2025-11-21

**Authors:** Rayssa Maitê Farias Nazário, Deborah Ribeiro Frazão, Leonardo Oliveira Bittencourt, Victoria Santos Chemelo, José Mário Matos‐Sousa, Beatriz Rodrigues Risuenho Peinado, Roberta Souza D’almeida Couto, Osmar Alves Lameira, João Daniel Mendonça de Moura, Jorddy Neves Cruz, Fabrício Mezzomo Collares, Rafael Rodrigues Lima

**Affiliations:** ^1^ Laboratory of Functional and Structural Biology, Institute of Biological Sciences Federal University of Pará Belém Pará Brazil; ^2^ Laboratory of Biotechnology, Embrapa Amazônia Oriental Belém Pará Brazil; ^3^ Laboratory of Dental Materials, School of Dentistry Federal University of Rio Grande do Sul Porto Alegre Rio Grande do Sul Brazil

**Keywords:** apical periodontitis, complementary therapies, *Copaifera reticulata* Ducke, endodontics, phytotherapy

## Abstract

**Aim:**

This study aimed to investigate the effects of copaiba oil–resin on induced apical periodontitis in rats.

**Methodology:**

A total of 24 male Wistar rats were divided equally into three groups (eight animals each): control (C), apical periodontitis (AP) and apical periodontitis with copaiba administration (AP + COP). The AP was induced by exposing the pulp chambers of the mandibular first molars to the oral environment. The openings were maintained for 28 days to allow lesion development. The AP + COP group received systemic administration of 200 mg/kg of copaiba oil–resin via intragastric gavage during the final 7 days of the AP induction period. The rats were then euthanised, and their hemimandibles were subjected to histopathological analysis to assess tissue preservation, histochemical staining with picrosirius red to evaluate collagen content, and microcomputed tomography to assess lesion volume and bone quality parameters. Statistical analyses were performed using a one‐way ANOVA, followed by Tukey's post hoc test for parametric data and the Kruskal–Wallis test for nonparametric data. The results are expressed as mean and standard error of the mean for parametric tests, and median and interquartile deviation for the nonparametric test.

**Results:**

The findings showed that copaiba oil–resin reduced lesion volume compared to the untreated group (*p* = 0.0261), as well as reducing the space between the bone trabeculae found in the AP group (*p* = 0.0063). Additionally, copaiba oil–resin preserved the collagen fibres, which were more degraded in the untreated group (*p* = 0.0009). Histopathological analysis showed that copaiba oil–resin reduced tissue damage, preserving a significant area of alveolar bone surrounding the lesion.

**Conclusions:**

These results indicate that copaiba oil resin has a promising adjunct therapeutic potential to reduce the bone loss caused by apical periodontitis and contribute to the maintenance of quality in the remaining bone.

**Abbreviations**
ABTS•^+^
: 2,2'‐Azino‐bis (3‐ethylbenzothiazoline‐6‐sulfonic acid)
AP: apical periodontitis group
AP + COP: apical periodontitis with copaiba administration group
BV: bone volume
BV/TV: bone volume percentage
C: control group

*C. reticulata*

: *Copaifera reticulata*
DPPH•: 2,2‐diphenyl‐1‐picrylhydrazyl
EDTA: ethylenediaminetetraacetic acid
Tb.N: trabecular number
Tb.Sp: trabecular spacing
Tb.Th: trabecular thickness

## 1 Introduction

Apical periodontitis is a common oral disease with a worldwide impact, affecting approximately 52% of adults, that usually involves at least one tooth (Tibúrcio‐Machado et al. 2021). This condition is marked by inflammation, primarily due to bacterial infections in the root canal (Wong et al. 2021), with anaerobic Gram‐negative bacteria often causing primary infections and anaerobic facultative Gram‐positive bacteria causing secondary infections (Wong et al. 2021). When these bacteria reach the apical third of the root canal and move beyond the foramen, they cause an extraradicular infection that triggers local inflammation and bone resorption in the apical region (Wang and Stashenko 1991).

Microbiome diversity and individual susceptibility present challenges for conventional endodontic treatments like instrumentation and sealing, especially in recurrent cases. These difficulties arise from various microbial and nonmicrobial factors, including extraradicular and intraradicular infections, as well as periodontal and prosthetic issues, all of which can lead to endodontic treatment failure (Karamifar 2020). From a clinical perspective, the consequences of untreated or recurrent apical periodontitis are significant. This condition contributes to persistent pain, systemic inflammation and progressive bone loss, potentially leading to tooth loss and a compromised quality of life (Wu et al. 2006). Given these outcomes, finding therapeutic strategies that not only control infection but also mitigate bone damage and inflammation is of paramount importance. As a result, the scientific community is increasingly exploring alternative therapies, using natural products, such as herbal extracts and flower essences, due to their antimicrobial and anti‐inflammatory properties and because they do not promote bacterial antibiotic resistance (Re et al. 1999; Vaou et al. 2021).

Copaiba, derived from the *Copaifera* genus, holds significant cultural and medicinal importance among traditional populations in Brazil where it is commonly used as a natural remedy, particularly among populations with limited access to first‐line medications (Da Rocha et al. 2022). Ethnobotanical records show that *Copaifera reticulata*, specifically, has been applied by indigenous groups for treating skin lesions, respiratory issues and gastrointestinal diseases (Da Silva et al. 2007). This genus is widely distributed across Africa, and Central and South America, with Brazil having the greatest diversity, featuring 26 species and 8 varieties (Da Trindade et al. 2018). Copaiba oil–resin is a liquid that contains a nonvolatile fraction made up of resin acids, including diterpenes, and a volatile fraction composed of sesquiterpenes (Da Trindade et al. 2018).

An analysis of the global scientific output on copaiba mapped the reported biological activities of copaiba and found that this oil–resin has been associated with various properties, such as anti‐inflammatory, antibacterial, antifungal and larvicidal effects (Frazão, Cruz, et al. 2023). This oil‐resin is rich in β‐caryophyllene, β‐bisabolene and α‐humulene, compounds known for their role in modulating inflammation and promoting tissue repair (Gertsch et al. 2008). Additionally, our group has demonstrated that experimental oral administration of copaiba oil–resin has no adverse effects on hepatic or renal function, supporting its safety profile within therapeutic dosage ranges (Alvarenga et al. 2020). Furthermore, this oil–resin exhibits biocompatibility with conjunctival cells, promoting cellular proliferation and migration, as well as facilitating biomineralisation through the induction of mineralisation nodule formation (Couto et al. 2020).

Recent study demonstrated that copaiba oil–resin effectively reduces inflammatory responses and decreases alveolar bone loss in a ligature‐induced periodontitis model in rats (Dos Santos et al. 2022). Furthermore, copaiba oil exhibited therapeutic effects on tissue repair in tongue lesions (Alvarenga et al. 2020; Teixeira et al. 2017). These findings highlight copaiba oil–resin as a viable and adjunctive therapeutic option. Although the literature presents several pieces of evidence on the effects of copaiba oil–resin in soft tissues, treating inflammatory and infectious conditions, studies focusing on its effects on bone tissue are limited (Frazão et al. 2024).

In this perspective, our experimental study aimed to investigate whether copaiba oil–resin per se is effective in reducing the bone damage and inflammatory response caused by apical periodontitis, without another endodontic therapy. By evaluating the therapeutic potential of copaiba oil–resin in this context, we aimed to provide perspectives on its applicability as an adjunctive therapy for this condition. The null hypothesis posited that copaiba oil–resin would not exert any beneficial effects on apical periodontitis.

## 2 Materials and Methods

### 2.1 Ethical Considerations

**FIGURE 1:**
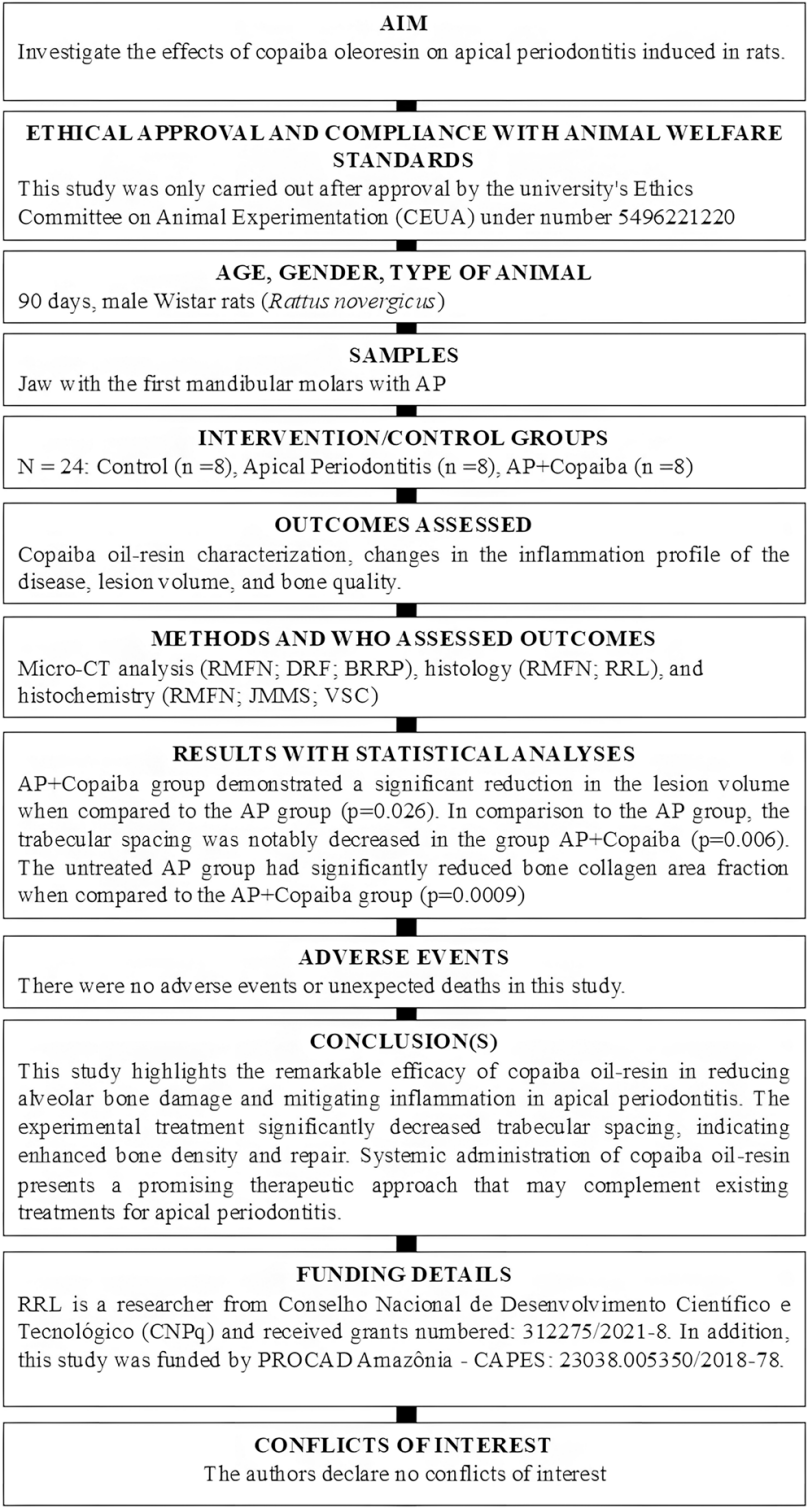
PRIASE flow chart.

This study was only carried out after approval from the university's Ethics Committee on Animal Experimentation (CEUA) under number 5496221220. We followed the Guide for the Care and Use of Laboratory Animals (National Research Council (US) Committee for the Update of the Guide for the Care and Use of Laboratory Animals 2011) and adhered to the ARRIVE 2.0 guidelines (Du Sert et al. 2020). In addition, the manuscript of this animal study has been written according to the Preferred Reporting Items for Animal Studies in Endodontology (PRIASE) 2021 guidelines (Nagendrababu et al. 2021). The flowchart of the study is shown in Figure 1, and the PRIASE checklist is included in Data S1.

### 2.2 Animals and Experimental Groups

Male albino rats ( *Rattus norvegicus* ) of the Wistar strain (*n* = 24; 90 days old) were sourced from the Federal University of Pará animal facility and acclimated. The sample size for each group was established using G*Power Software (Statistical Power Analyses 3.1.9.2), based on a previous study (Minhoto et al. 2021). The rats were kept in polypropylene nursery cages with unlimited access to food and distilled water, a 12‐h light/dark cycle (lights on at 7 a.m.), and temperature control (25°C ± 1°C). The rats were randomly assigned to cages housing four animals each, with each cage labelled accordingly. They were divided into three groups: control (C; *n* = 8), apical periodontitis (AP; *n* = 8) and apical periodontitis with copaiba administration (AP + COP; *n* = 8) (Figure 3). The oil–resin dosage was established following the methods by Teixeira et al. (2017), in compliance with OECD Guide 425/2008 for toxicity testing. We prepared an emulsion with a 200 mg/kg/day dosage, with the fluid phase containing a surfactant composed of 5% Tween 20 and saline solution (Teixeira et al. 2017). The researchers could not blind the allocation and administration stages due to the visible presence of copaiba oil–resin during gavage. However, blinding was used in the histopathological analysis, where the examiner was unaware of group allocation when evaluating samples. During the experiment, the animal care team was made up of experts with advanced degrees (MSc and PhD) in health‐related disciplines, and certifications in animal training and welfare.

### 2.3 Copaiba Oil–Resin Characterisation

**FIGURE 2:**
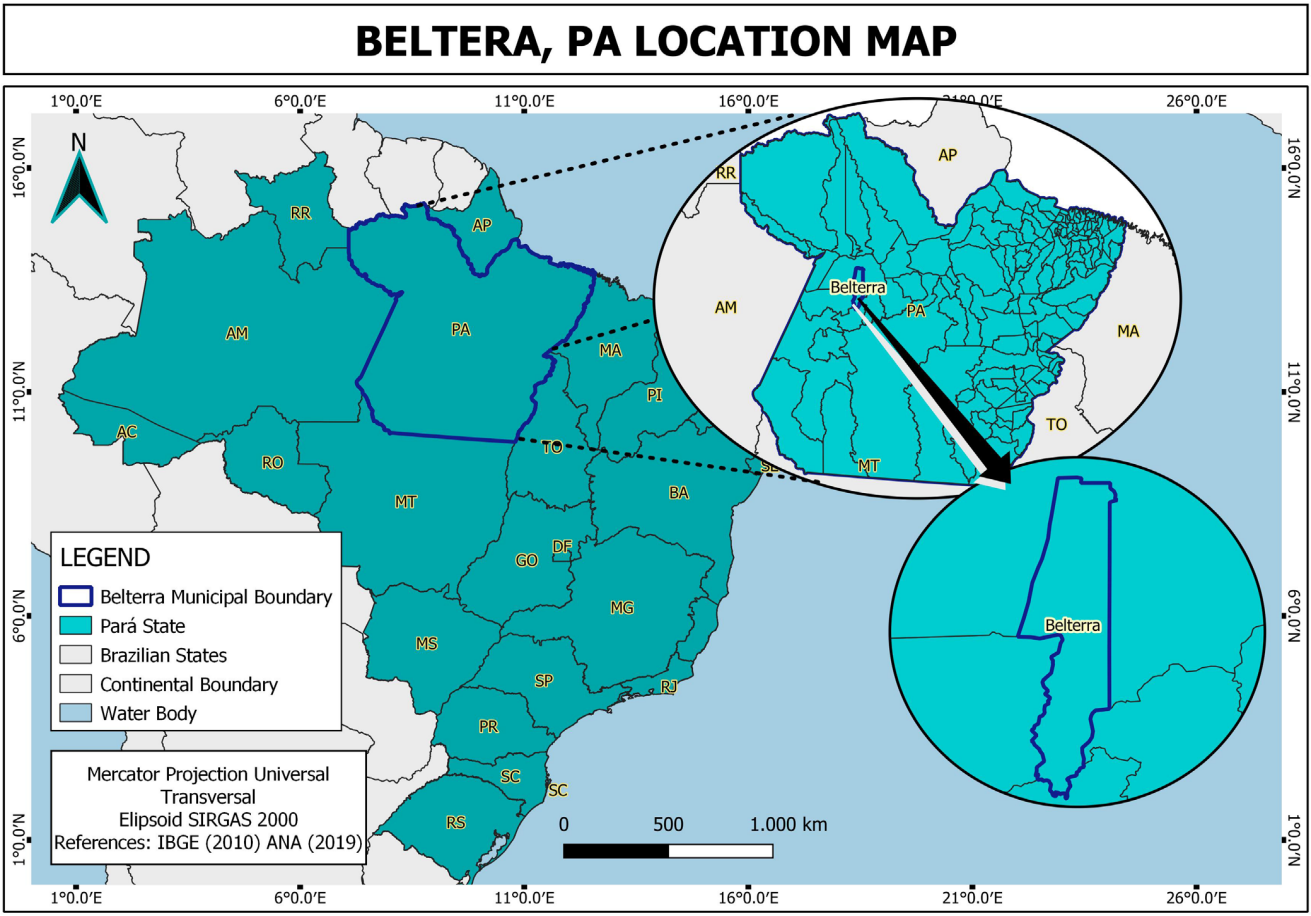
Geographic location of Belterra in Pará, Brazil.

The copaiba oil–resin was obtained and provided by the Brazilian Agricultural Research Company (*Empresa Brasileira de Pesquisa Agrícola*—EMBRAPA) (Ribeiro et al. 2024). This study is registered under protocol number AC548DA in the National Management System for Genetic Heritage and Associated Traditional Knowledge platform from the Brazilian Ministry of Environment. The oil–resin was obtained from a 30‐year‐old tree located in the experimental campus of EMBRAPA, in the district of Belterra, State of Pará, Brazil (locality KM 67, Flona do Tapajós, BR 163). Geolocation data from the extraction site was used to create a location map (Figure 2), generated using Google Earth platform. We also employed the Universal Transverse Mercator projection with an ellipsoidal correction at a scale of 1:100 000 000.

**FIGURE 3:**
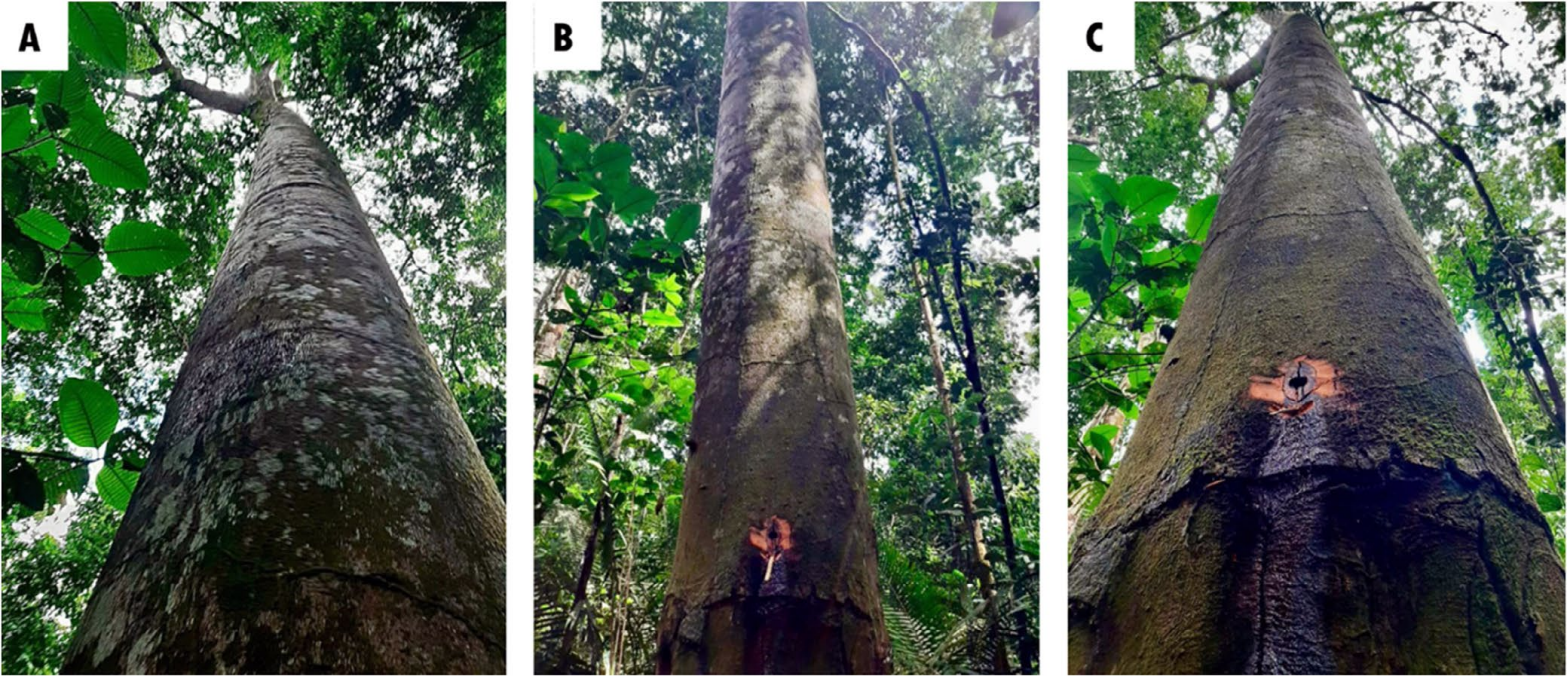
Three images depict the copaiba tree (*Copaifera reticulata* Ducke). (A) The trunk of the Copaiba tree. (B) A specific area of the trunk, marking the site where the oil–resin is collected. (C) Provides a magnified view of the collection site, showing the Copaiba oil–resin flowing down the tree trunk. The photo credits are attributed to the authors.

The oil–resin was obtained by artificial exudation, through holes made in the trunk of the tree (Figure 3); after the oil–resin harvest, both holes were plugged. The oil–resin was stored in darkness, without oxygen or heat, to stabilise its volatile metabolites. The species was identified by Regina Celia Viana Martins da Silva, and its exsiccata are deposited in the Herbarium of the *Instituto Agronômico do Norte*, which is under the responsibility of EMBRAPA, under the code ‘IAN:183939’ (Ribeiro et al. 2024).

The chemical composition of the oil–resin was obtained by chromatographic analysis according to a protocol published by Sousa et al. (2011). Gas chromatography–mass spectrometry (GC–MS) equipment, in the INCOSE Finnigan Mat XL system, was used, equipped with a silica capillary column DB5MS (30 cm × 0.25 mm, 0.25 μm film thickness), with the following operating conditions: carrier gas: helium at linear velocity of 32 cm/s (measured at 100°C); type of injection: ‘splitless’ (1 μL of a solution 2:1000 hexane); injector temperature and detector: 250°C; temperature program: 60°C–240°C (3°C/min); MS: electron impact, 70 eV; source temperature of ions and connecting pieces: 180°C. The components were identified by comparing their mass spectra and retention indices (RIs) with those of standard substances, the existing system libraries and with literature data (Adams 2007). The RIs were obtained using the homologous n‐alkanes. The quantification of components was obtained by GC, HP5890‐II, equipped with a flame ionisation detector (FID) and coupled to a HP3396 integrator‐II under the same operating conditions, except that the carrier gas was hydrogen. Using this procedure, it was possible to obtain copaiba oil–resin samples with high purity. GC–MS analysis revealed that the main metabolites of the copaiba oil–resin were β‐caryophyllene (37.3%), β‐bisabolene (14.5%) and trans‐α‐bergamotene (9.0%) (Guimarães‐Santos et al. 2012).

### 2.4 Antioxidant Capacity of the Copaiba Oil–Resin

The antioxidant capacity of *Copaifera reticulata* Ducke's oil–resin was evaluated using the ABTS•^+^ (2,2′‐Azino‐bis [3‐ethylbenzothiazoline‐6‐sulfonic acid]) and DPPH• (2,2‐diphenyl‐1‐picrylhydrazyl) assays. The antioxidant capacity of Copaiba oil–resin was assessed by comparing it to Trolox (6‐hydroxy‐2,5,7,8‐tetramethylchromono‐2‐carboxylic acid), a water‐soluble analogue of vitamin E. In addition, to illustrate the directly correlated values, we calculated the antioxidant activity using standard equations (ABTS•^+^ *y* = 0.455*x* + 0.0002 *R* ^2^ = 0.998; DPPH• *y* = 0.2261*x* − 0.0094 *R* ^2^ = 0.9831). The analysis of the oil was performed in triplicate (*n* = 3).

### 2.5 DPPH

This approach was used to determine the ability of *C. reticulata* Ducke oil‐resin to block the 1,1‐diphenyl‐2‐picrylhydrazyl (DPPH•) radical, a violet chromophore, resulting in the creation of the hydrogenated DPPH product, that is yellow or colourless (Blois 1958). To evaluate antioxidant capacity, we initially measured the absorbance of a 0.1 mM DPPH• solution (2,2‐diphenyl‐1‐picrylhydrazyl) diluted in ethanol. Subsequently, we combined 0.6 mL of the DPPH• solution, 0.35 mL of distilled water, and 0.05 mL of the sample, and incubated the mixture in a water bath at 37°C for 30 min. We measured the absorbance at 517 nm using an Nm Kasvi spectrophotometer. Trolox served as the reference for the calibration curve, with results expressed in mM and compared to a Trolox standard (1 mM).

### 2.6 ABTS

The ABTS•^+^ radical scavenging assay followed the protocol by Miller et al. (1993), adapted by Re et al. (1999). To generate ABTS•^+^, we incubated 7 mM ABTS with 140 mM potassium persulfate (K_2_O_8_S_2_) at room temperature for 16 h, protected from light. The resulting solution was diluted with phosphate‐buffered saline to an absorbance of 0.700 (±0.02) at 734 nm.

We used Trolox as the standard for the calibration curve. To evaluate the antioxidant capacity, we transferred 2.97 mL of the ABTS•^+^ solution to the cuvette and measured the absorbance at 734 nm. Next, 0.03 mL of the sample was introduced into the cuvette containing the ABTS•^+^ radical, and after a 5‐min incubation period, the second reading was obtained. The results were expressed in mM, and the values obtained for the samples were compared to the Trolox standard (1 mM).

### 2.7 Apical Periodontitis Induction

Experimentally induced apical periodontitis was carried out based on the studies of Aksoy et al. (2019), Frazão, Santos Mendes, et al. (2023), and Sehirli et al. (2019). The animals were anaesthetised intraperitoneally using a combination of 2% xylazine (8 mg/kg) and 10% ketamine (90 mg/kg) and positioned on an operating table. A #1/4 carbide burr was used to expose the pulp of the left and right lower first molars. Pulpal bleeding confirmed root canal access. The exposed teeth remained open to the oral environment for 28 days to facilitate the development of apical lesions. To mitigate pain and discomfort, we administered dipyrone subcutaneously at a dosage of 100 mg/kg daily for 3 days.

### 2.8 Copaiba Administration and Sample Collection

The experimental timeframe consisted of 28 days, with apical periodontitis induced on day 0. Throughout this period, the lesion remained exposed to the oral cavity of the animals. Starting on day 21, the animals of the group AP + COP received copaiba oil–resin daily, for 7 days, at 24‐h intervals. The oil–resin was administered via intragastric gavage, adjusting the dose to 200 mg/kg based on each animal's weight, which we monitored regularly. The C and AP groups received an equivalent oral volume of distillate water via gastric gavage, similar to the AP + COP group. This activity was registered on the National System for the Management of Genetic Heritage and Associated Traditional Knowledge (SISGEN) platform of the Ministry of the Environment (Government of Brazil) under number AC548DA.

On Day 28, the animals from all groups (C, AP, AP + COP) were anaesthetised with ketamine hydrochloride (90 mg/kg) and xylazine hydrochloride (8 mg/kg). After the loss of corneal eye reflex, the rats were perfused through the left ventricle with a 0.9% saline solution containing 1% heparin, followed by 4% formaldehyde. Subsequently, mandibular samples were collected, and after gingival dissection, the hemimandibles were post‐fixed and immersed in 4% formaldehyde for the subsequent methodologies. One hemimandible was used for microtomographic analysis, and the other hemimandible for histological analysis.

### 2.9 Microcomputed Tomography Analysis (Micro‐CT)

Radiographic images of the hemimandible were acquired by employing a 360° rotation and operating at an intensity of 70 kV/100 mA. This process, using microtomography (MicroCT.SMX‐90 CT; Shimadzu Corp., Kyoto, Japan), resulted in 541 radiographic images covering the entire length of the mandible of each sample. Subsequently, we reconstructed the radiographic images with a voxel size of 10 μm and a resolution of 1024 × 1024 using inspeXio SMX‐90CT software (Shimadzu Corp., Kyoto, Japan). Finally, we converted the obtained data to the Digital Imaging and Communications in Medicine (DICOM) format.

CTAn software (V1.15.4.0; Bruker, Kontich, Belgium) was utilised to reconstruct the alveolar bone area for data analysis. In this reconstruction, the positioning of the hemimandibles was standardised to enhance the visibility of the space of the periodontal ligament in the sections. Following the method described by Chen et al. (2019), the reconstructed volume of interest (VOI) was delineated, encompassing the ligament space and the surrounding area of bone destruction around the roots. Subsequently, the examiner, who was blinded and calibrated, manually outlined the volume of the destroyed area. This process began at the mesial root and extended to the distal root of the lower first molar. For each root assessed, the VOI started with the first coronal section of the mesial root, surrounded by the bone crest, proceeded to the distal region and concluded upon encountering the second lower molar.

To assess the alveolar bone quality, CTAn software (V1.15.4.0; Bruker, Kontich, Belgium) was utilised to analyse a set of 220 images of the bone region surrounding the first lower molar. The region of interest was defined as the alveolar bone encasing the molar roots. For the analysis, the calibrated examiner manually delineated the bone in each coronal plane, starting from the proximal point of the mesial root and extending to the distal point of the distal root. CTAn software employed a threshold range of 31–71 in the grey scale of each image to distinguish between cortical bone, trabecular bone and bone marrow. Quality was evaluated based on the following parameters: bone volume (BV), bone volume percentage (BV/TV), trabecular thickness (Tb.Th), trabecular number (Tb.N) and trabecular spacing (Tb.Sp). These metrics were measured in the bone unaffected by the lesion (Frazão, Santos Mendes, et al. 2023).

### 2.10 Sample Processing and Histological Analysis

The hemimandible was collected and post‐fixed in 4% formaldehyde for 24 h. Following this, we demineralised the samples in 10% EDTA for 90 days. The specimens underwent dehydration in alcohol, diaphanisation in xylene and embedding in Paraplast (Sigma‐Aldrich, USA). Once embedded, the samples were sectioned mesiodistally at a thickness of 5 μm, using a Leica RM 2045 microtome (Leica Microsystems, Nussloch, Germany), and mounted on individual slides. The histological sections for histopathological analysis were stained with haematoxylin and eosin and then, photomicrographs were taken with a digital colour camera (C‐mount, DS‐Fi3; Nikon, Tokyo, Japan), coupled to an optical microscope (Eclipse Ci‐L; Nikon, Tokyo, Japan; using 10× and 40× objective lenses). Qualitative analysis was performed by a blinded histopathologist, who assessed the tissue preservation profile from semi‐serial sections along the entire mandible. The state of preservation comprised the degree of integrity of the interradicular and periapical alveolar bone, as well as the preservation of the periodontal ligament and cementum (Frazão, Santos Mendes, et al. 2023).

### 2.11 Histochemical Analysis

**FIGURE 4:**
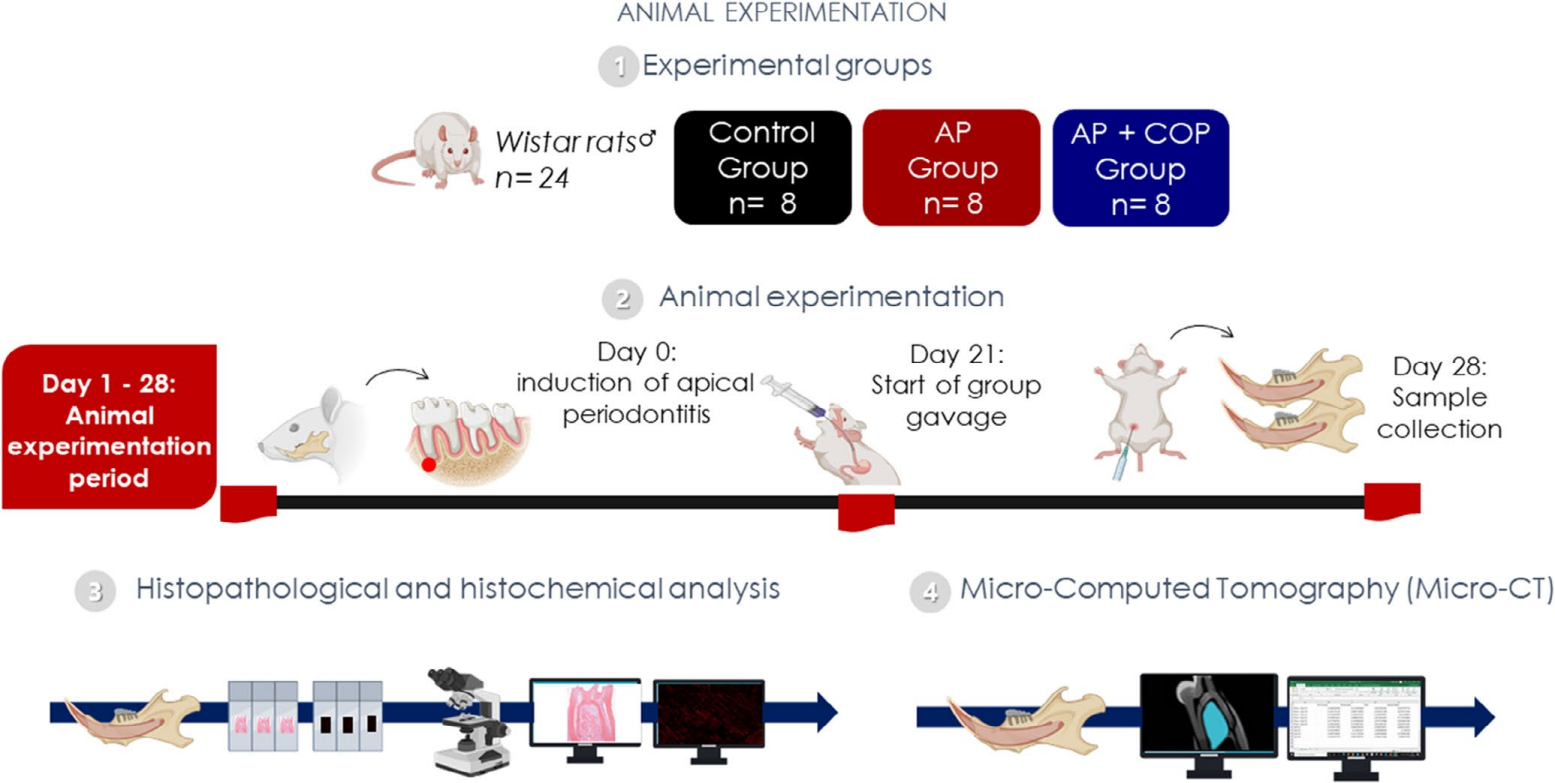
Methodological figure representing the experimental stages, which were divided into four stages. (1) the animals were randomised into the sample groups, and the Copaiba oil–resin was characterised. (2) the in vivo experimentation period was carried out, comprising the induction of the periapical lesion, copaiba oil–resin gavage, and sample collection. (3) the histopathological and histochemical procedures were carried out. (4) the microcomputed tomography (micro‐CT) analyses were conducted.

To assess the collagen Type 1 content in the remaining alveolar bone tissue, we stained sections of the alveolar bone with picrosirius red and observed them under a polarised light microscope at 40× optical lens (Junqueira et al. 1979; Matos‐Sousa et al. 2024). Using ImageJ, we estimated the collagen area for each sample by calculating the arithmetic mean of threshold percentages from five fields/sections (Matos‐Sousa et al. 2024). The area of bone collagen was measured as a percentage (%). All experimental procedures are summarised in Figure 4.

### 2.12 Statistical Analysis

The assessment of normality was conducted using the Shapiro–Wilk test. Statistical analyses were performed with one‐way ANOVA followed by the Tukey post hoc test to assess bone quality and lesion volume (parametric data) and the results were presented as mean ± standard error. Collagen fibre analysis with PicroSirius Red was performed with the Kruskal–Wallis test and reported as median and interquartile deviation (nonparametric data). The test power was determined based on the difference between two means using OpenEpi (version 2.3.1), with a type I error set at 5% and a minimum power of 80%. Statistical significance was defined as *p* < 0.05. GraphPad Prism 9.0 software (GraphPad, San Diego, CA, USA) was employed for all statistical analyses.

## 3 Results

### 3.1 Antioxidant Capacity of Copaiba Resin Oil

The DPPH• values were 0.812 ± 0.032 mM, indicating the activity of copaiba oil–resin in the presence of the radical and its high antioxidant capacity. Furthermore, the ABTS•^+^ levels were 0.163 ± 0.004 mM. These results suggest that copaiba oil–resin has significant potential to combat oxidative stress.

### 3.2 Copaiba Oil–Resin Effectively Reduced the Lesion Volume Associated With Apical Periodontitis

**FIGURE 5:**
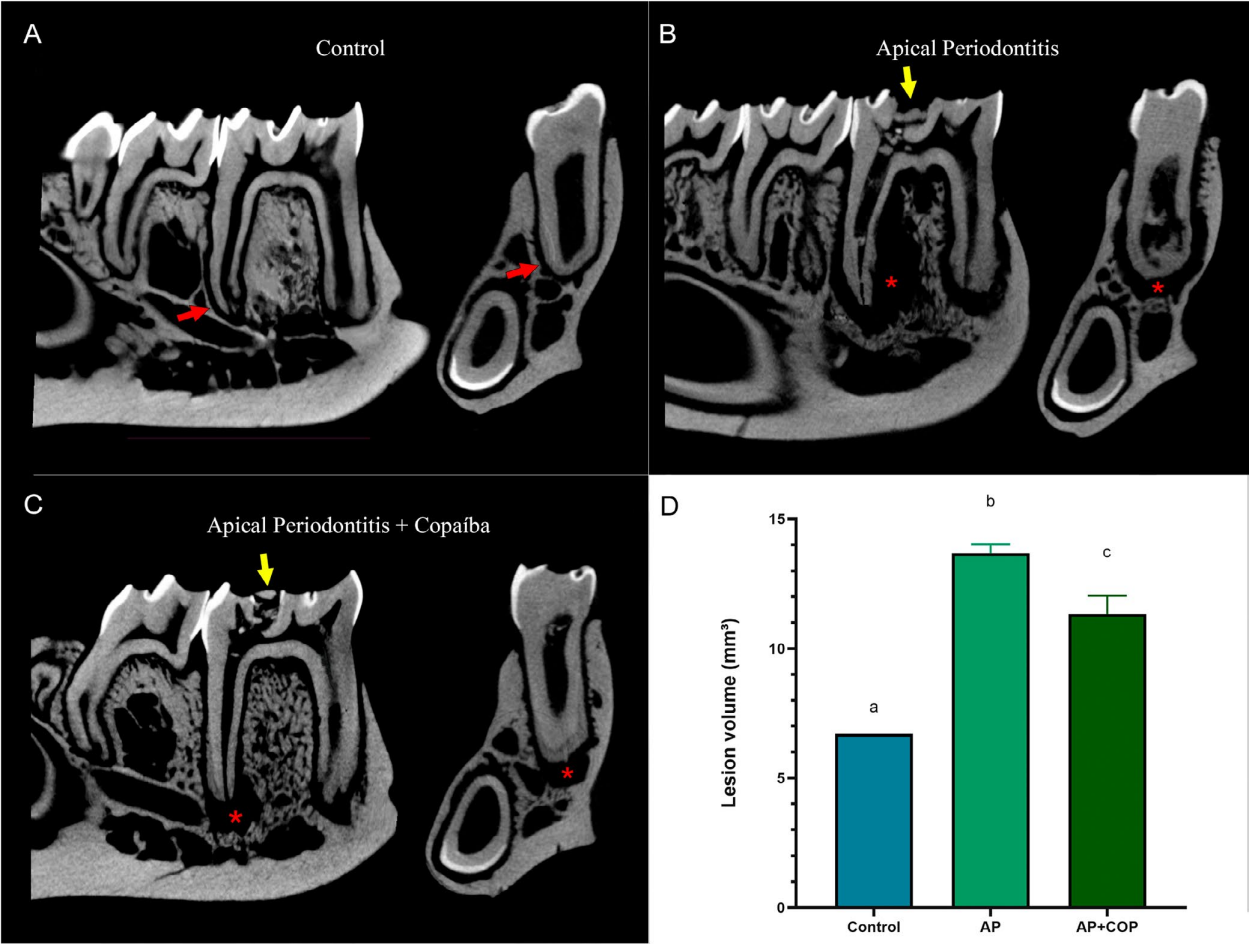
Representative micro‐CT scans of the lower first molar region in sagittal and coronal sections of the experimental groups. (A) the micro‐CT of the control group, with red arrows indicating the periodontal ligament space. (B) the apical periodontitis group, the yellow arrow shows the site of the pulp access surgery to induce the endodontic lesion, and red asterisks indicate the volume of the apical lesion. (C) the group with apical periodontitis with copaiba administration, the yellow arrow shows the site of the pulp access surgery, and red asterisks indicate the volume of the apical lesion. (D) the results of the lesion volume (mm^3^) in the control, apical periodontitis, and apical periodontitis with copaiba administration groups. The results are presented as the mean ± standard error of the mean. The data were analysed using one‐way ANOVA and Tukey's post hoc test. Statistical significance (*p* < 0.05) was indicated by different letters (a, b and c), with equal letters indicating no statistical difference and different letters indicating a statistical difference between the groups.

The apical periodontitis group showed a significantly larger lesion volume in comparison with the control group (14 ± 0.36 mm^3^ vs. 6.7 ± 0.58 mm^3^; *p* < 0.0001), marked by an increased volume in the ligament space and bone loss around the roots. On the other hand, the AP + COP group demonstrated a significant reduction in lesion volume when compared to the group with untreated lesions (11 ± 0.69 mm^3^ vs. 14 ± 0.36 mm^3^; *p* = 0.0261) (Figure 5).

### 3.3 Copaiba Oil‐Resin Modified the Trabecular Spacing in Bone Quality Analysis

**FIGURE 6:**
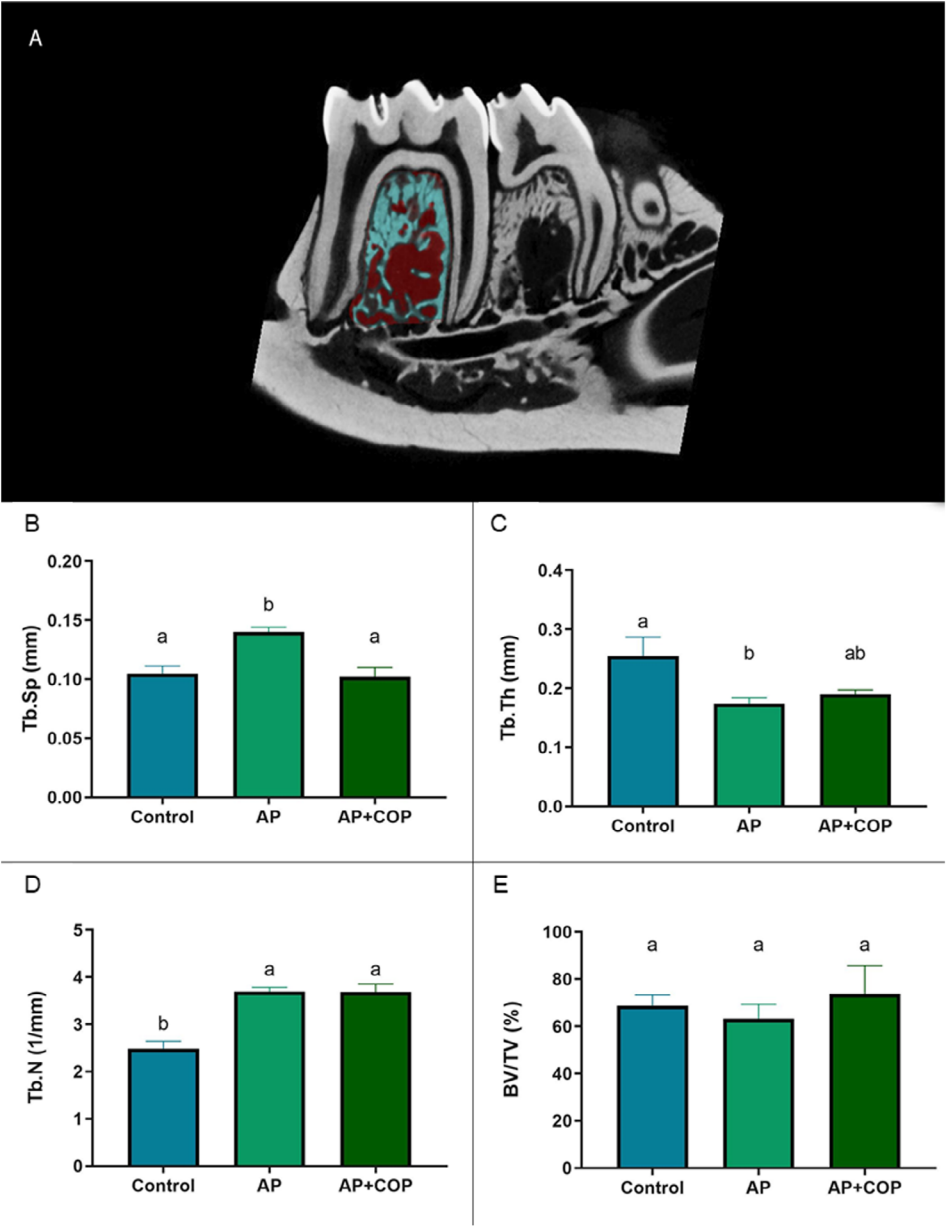
(A) red and blue markings show the region of interest defined in CTan software for evaluating bone quality parameters. (B) the trabecular spacing parameter (Tb.Sp); (C) the trabecular thickness parameter (Tb.Th); (D) the number of trabeculae parameter (Tb.N). (E) the percentage ratio between bone volume and tissue volume (BV/TV). The one‐way ANOVA with Tukey post hoc test was utilised (*p* < 0.05). Statistical significance (*p* < 0.05) was indicated by different letters (a and b), with equal letters indicating no statistical difference and different letters indicating a statistical difference between the groups.

The region of interest defined in CTan software is shown in Figure 6A. In comparison to the apical periodontitis group per se (0.14 ± 0.0082 mm), the trabecular spacing (Tb.Sp) was notably decreased in the AP + COP group (0.10 ± 0.018 mm; *p* = 0.0063). In addition, the AP + COP exhibited no significant distinction from the control group (0.11 ± 0.015 mm; *p* = 0.9404) (Figure 6B).

The apical periodontitis with copaiba administration group (0.19 ± 0.071 mm) showed no statistical difference in trabecular thickness (Tb.Th) compared to either the control (*p* = 0.13) or apical periodontitis group (*p* = 0.56). Nevertheless, the AP group (0.16 ± 0.014 mm) presented a reduction in Tb.Th compared with the C group (0.26 ± 0.032 mm; *p* = 0.01) (Figure 6C).

There was no difference in trabecular number (Tb.N) between the apical periodontitis and apical periodontitis with copaiba administration groups (3.7 ± 0.084 vs. 3.8 ± 0.17; *p* = 0.9985) (Figure 6D). However, both groups exhibited a significant increase compared with the control group (2.5 ± 0.17; *p* = 0.0002). Additionally, no significant differences were observed in the percentage of bone volume to tissue volume ratio (BV/TV) among the groups (C: 69% ± 1.9% vs. AP: 63% ± 3.1% vs. AP + COP: 74% ± 5.4%; *p* = 0.1783) (Figure 6E).

### 3.4 Copaiba Oil–Resin Reduced the Magnitude of the Damage and the Inflammatory Response

**FIGURE 7:**
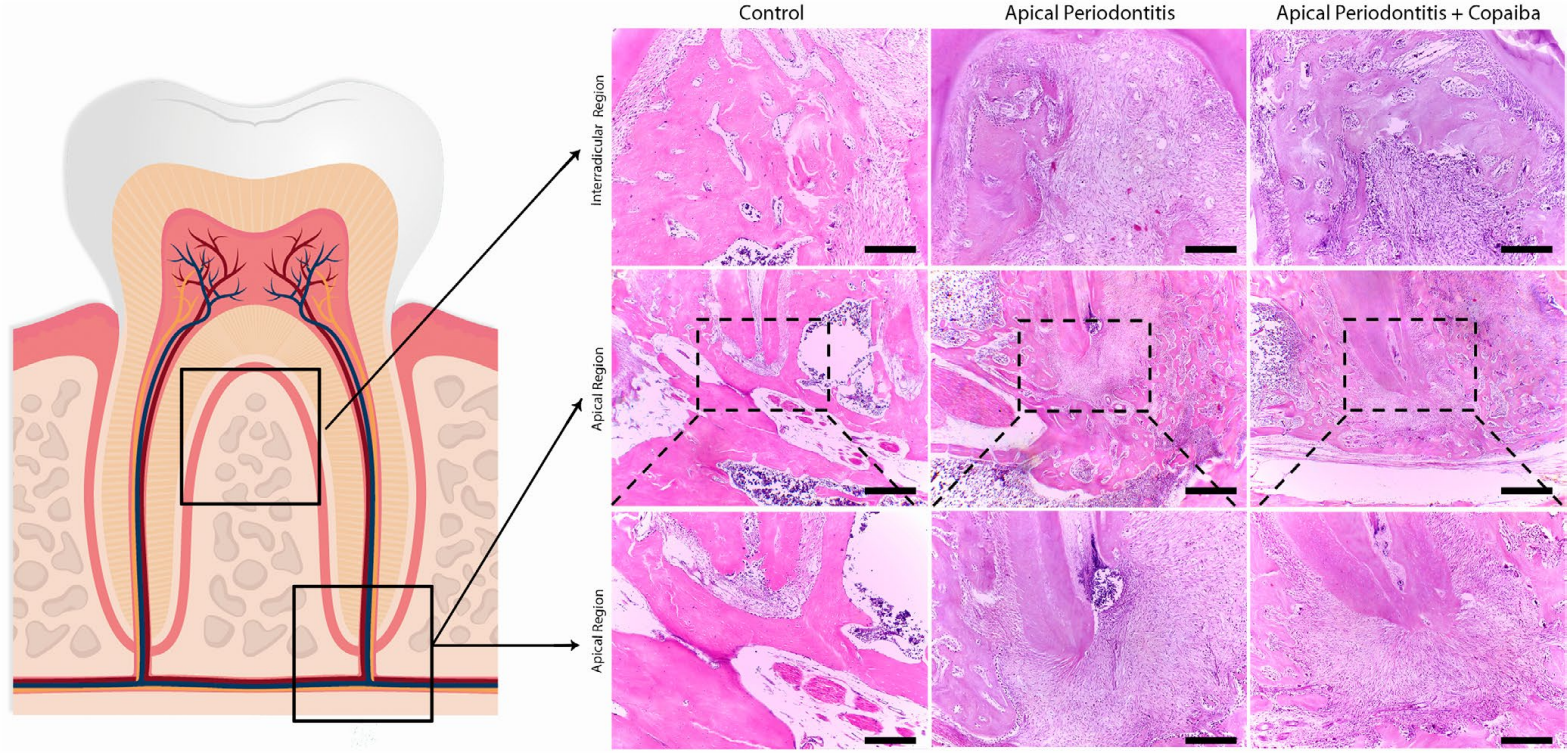
Photomicrographs of the experimental groups. The left panel (A) depicts the first molar, with squares indicating the region of each micrograph, namely the interradicular region and the apical region of the tooth. The first, second and third columns show the control (B–D), apical periodontitis (E–G) and apical periodontitis with copaiba administration (H–J) groups, respectively. The first row represents the furcation area (B, E, H) taken with the objective lens 10× (50 μm‐scale), and the second (C, F, I) and third (D, G, J) rows indicate the apical region taken with the objective lenses 4× (100 μm‐scale) and 10× (50 μm‐scale), respectively. The dashed lines outline the apical region in all groups. In the AP group, this area shows pronounced inflammatory infiltrate (arrow head), which is reduced in the AP + COP group (arrow head). In contrast, the apical region in the control group shows no signs of inflammation.

Histopathological observations revealed uninterrupted bone structure in the control group (Figure 7B–D). This was observed by preserved bone trabeculae in the interradicular (Figure 7B) and periapical regions (Figure 7C,D), as well as the integrity of the periodontal ligament and cementum (Figure 7C,D). In contrast, the group with untreated periapical lesions (Figure 7E–G) exhibited tissue alterations and substantial bone resorption in the interradicular region (Figure 7E). On the other hand, the group that received copaiba oil‐resin (Figure 7H–J) showed preservation of alveolar bone (Figure 7H). In the periapical region, the untreated group showed an active periapical lesion marked by discontinuity of the alveolar bone and rupture of the periodontal ligament on Day 28 of the experiment (Figure 7F,G). These findings align with the experimental timeline in which inflammation becomes chronic. Conversely, the AP + COP group showed minimisation of the periapical lesion and greater bone preservation when compared to the untreated group (Figure 7I,J).

### 3.5 Copaiba Oil–Resin Preserves Bone Collagen Fibres Against the Inflammatory Process

**FIGURE 8:**
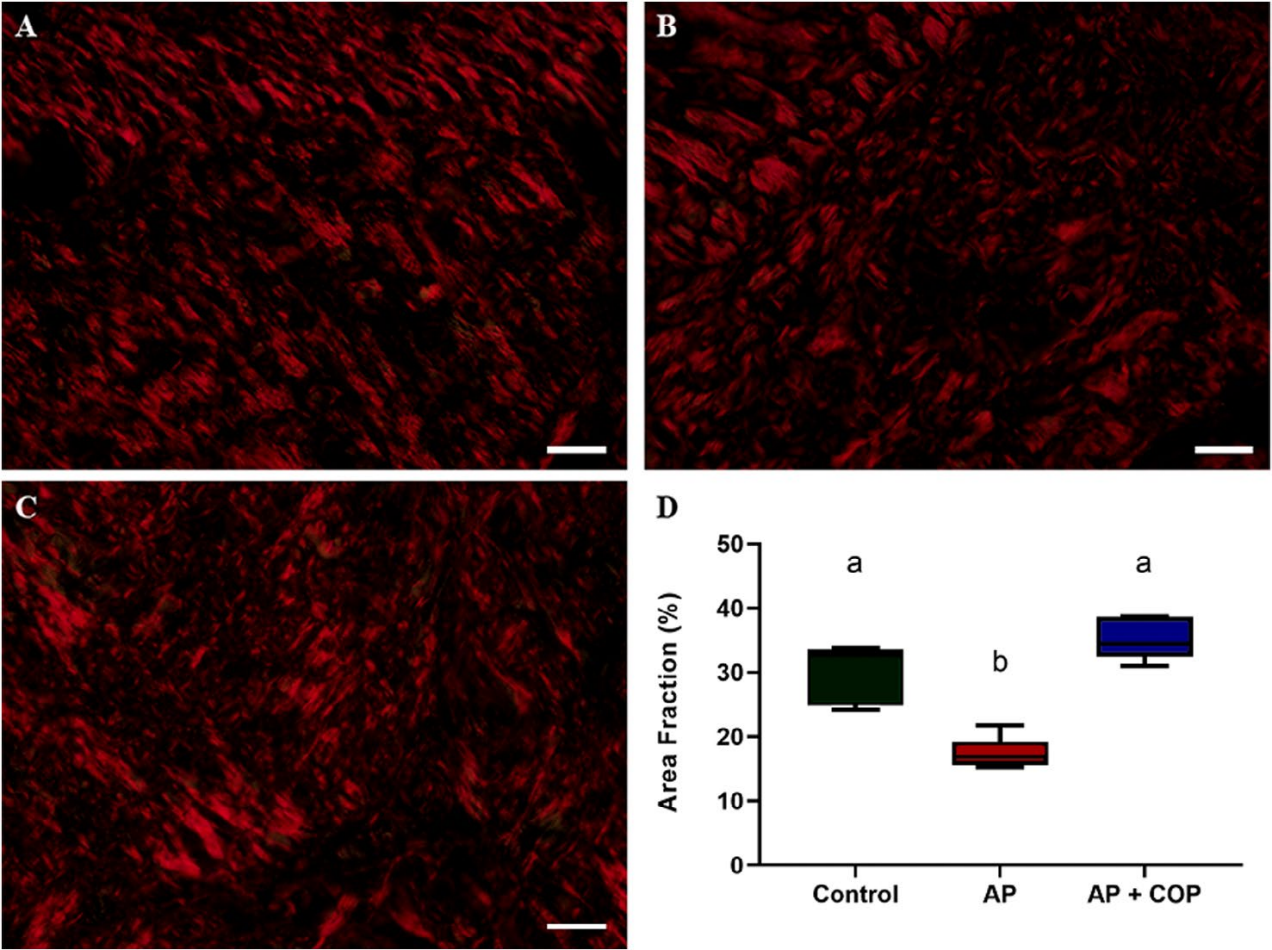
Representative photomicrographs of collagen content analysis using picrosirius red staining. (A–C) present the control, apical periodontitis and apical periodontitis with copaiba administration groups, respectively. The white arrows indicate the collagen fibre stained by Picrosirius Red. (D) shows the graph of the percentage of bone collagen area (%). Scale bar: 20 μm. Data analysed with the Kruskal–Wallis test. Statistical significance (*p* < 0.05) was indicated by different letters (a and b), with equal letters indicating no statistical difference and different letters indicating a statistical difference between the groups.

The analysis of collagen fibres revealed that the untreated apical periodontitis group (median: 16.80%; interquartile range: 15.61–19.13) presented a reduced bone collagen area fraction in the remaining bone when compared to the control group (median: 32.52%; interquartile range: 24.87–33.62; *p* = 0.0426). However, the administration of copaiba oil–resin resulted in a significantly higher bone collagen area fraction (median: 34.45%; interquartile range: 32.47–38.63) compared with the AP group (*p* = 0.0009), in the remaining bone. No significant differences were observed in the bone collagen area fraction when comparing the AP + COP to the control group (*p* = 0.6677) (Figure 8).

## 4 Discussion

This study demonstrates that copaiba oil–resin reduces the extent of bone destruction caused by apical periodontitis in rats, as evidenced by precise lesion volume measurements and histological analysis. In fact, the systemic administration of copaiba oil–resin attenuated the repercussions of apical periodontitis on the alveolar bone, by preserving trabecular spacing in the remaining alveolar bone adjacent to the lesion. Furthermore, the collagenous element of the bone's organic matrix, collagen fibre, remained preserved, supporting the tissue integrity and homeostasis. Thus, our findings highlight the potential of copaiba oil–resin as a biologically active adjunct in modulating tissue response during apical periodontitis, including bone and immunological modulation, offering new perspectives for therapeutic strategies in endodontic pathologies, such as periapical conditions.

The pathogenesis of apical periodontitis begins with bacterial invasion through breaches in the protective mineralised tissue layers, leading to pulpal inflammation and periradicular disease. The severity of the condition can vary, with some cases being asymptomatic while others involve severe pain and significant complications (Wong et al. 2021; Tibúrcio‐Machado et al. 2021). In the current study, we observed significant bone resorption at 28 days, consistent with previous reports that describe a predictable progression in rats, with an initial increase in lesion volume after between 14 and 21 days, followed by a chronic phase marked by substantial bone loss (Wang and Stashenko 1991). This timeline aligns with established endodontic lesion development observed in micro‐CT analyses and histological examinations (Wang and Stashenko 1991). The molecular mechanisms that drive the evolution of periapical disease, as well as the orchestration of the associated inflammatory response, have important significance for understanding and treating periapical disorders (Cintra et al. 2021; Frazão, Santos Mendes, et al. 2023).

In the antioxidant capacity analysis, we found that the copaiba oil–resin used in this study exhibited high antioxidant activity, which is capable of mitigating oxidative stress, a key contributor to tissue degradation in apical periodontitis. The antioxidant activity of copaiba oil–resin showed distinct values in the ABTS and DPPH assays, with relatively lower levels in the ABTS assay. This discrepancy is commonly reported in the literature and can be attributed to the structural and reactivity differences of the radicals involved in each method (Dos Santos et al. 2024; Munteanu and Apetrei 2021). The ABTS•^+^ radical is more hydrophilic in nature and can interact to a limited extent with mostly lipophilic compounds, such as terpenes and diterpenes present in copaiba, due to steric and kinetic barriers. On the other hand, the DPPH• radical, with its lipophilic nature and high solubility in organic solvents, has a greater affinity for these substances, resulting in more pronounced radical scavenging activity (Çelik et al. 2010). Thus, the reduced values observed in the ABTS assay do not indicate a lack of antioxidant potential, but rather reflect the specific chemical reactivity profile of the oleoresin. Similar results have been reported for other terpenoid‐rich essential oils and resins, reinforcing the need to consider the chemical nature of the constituents and the complementarity between different assessment methods when interpreting antioxidant capacity (Franco et al. 2022).

Copaiba was systemically administered to the animals on the 21st day after the lesion induction. According to the literature, this timeframe correlates to the established presence of an endodontic lesion, as observed through micro‐CT examination (Balto et al. 2000). Studies utilising micro‐CT identified significant volumes of periapical bone loss over the 21‐day progression of apical periodontitis, findings that align closely with the histological analyses (Balto et al. 2000; Wang and Stashenko 1991). Furthermore, the data showed enhanced bone structure retention, notably in terms of trabecular spacing (Tb.Sp), which is the average distance between adjacent trabeculae and reflects the level of bone loss or thinning within the trabecular network. A greater Tb.Sp value implies more gaps between trabeculae, indicating lower bone density and increased bone porosity.

There is currently no indication that copaiba oil–resin from the species used in the present study, *C. reticulata* Ducke, interacts directly with bone cells; that is, it is not known whether conserving lesion volume causes an increase in osteoblast activity, a decrease in osteoclastogenesis, or both. However, the main component of this oil‐resin, β‐caryophyllene, has been demonstrated to induce osteoblastic mineralisation, inhibit adipogenesis, and reduce osteoclastogenesis in mouse bone marrow cell cultures in vitro (Yamaguchi and Levy 2016). This metabolite, present in botanicals that are typically ingested daily and recognised by the FDA as a food additive, operates as a selective CB2 agonist and has anti‐inflammatory properties in animals. The comparable effects of copaiba may be attributed to lower TNF‐α and IL‐1β production associated with opioid receptors' signalling pathways (Yamaguchi and Levy 2016).

Histological examination of the periapical lesion site demonstrated that copaiba oil–resin administration minimised the damage in the bone surrounding the apical lesion, and consequently, the lesion volume. This preservation was closely linked to the maintenance of trabecular spacing, as validated by microtomography data, and had a major impact on collagen deposition and tissue organisation. These findings align with previous reports, that reported significantly better collagen organisation in copaiba‐treated wounds compared to control and corticoid groups, as shown by picrosirius red staining and birefringence scoring (Alvarenga et al. 2020).

Copaiba may serve an important role in collagen preservation, since it reduces inflammation, which contributes to collagen degradation (de Almeida et al. 2021). Furthermore, Copaiba appears to improve tissue healing and repair mechanisms, possibly via activating fibroblasts, the primary cells responsible for collagen formation (de Almeida et al. 2021). This provides a more favourable environment for collagen synthesis and preservation. Furthermore, our findings show that copaiba not only prevents excessive collagen accumulation but also keeps it structurally intact, even in the presence of fibrous repair tissue. These combined results highlight copaiba's potential for maintaining bone tissue, reducing inflammation and optimising collagen deposition inside periapical lesions, ultimately contributing to the support and integrity of the damaged bone.

Our findings align with a previous study from our group, that demonstrated the therapeutic potential of copaiba oil–resin in oral inflammatory conditions involving bone loss. The study showed that copaiba administration reduced inflammation and preserved alveolar bone structure in a ligature‐induced periodontitis model in rats (Dos Santos et al. 2022). Similarly, our current evaluation observed significant anti‐inflammatory effects and overall preservation of alveolar bone in the periapical region, evidenced by reduced lesion volume in the copaiba group. However, while both studies showed beneficial effects on bone microarchitecture, some specific parameters, such as Tb.Th, BV/TV and Tb.N demonstrated different levels of significance between the two studies. These discrepancies likely stem from differences in the regions of interest (ROI). While the current study analysed the entire alveolar bone surrounding the periapical lesion, the previous study focused on interradicular bone below the furcation in periodontitis. Distinct bone compartments, subjected to varying biomechanical stresses and microenvironments, may drive these parameter‐specific outcomes. Notably, the current study expands on prior findings by demonstrating that copaiba significantly preserved bone collagen content, a parameter that was not assessed in the Dos Santos et al. (2022) study. Collectively, both studies underscore the protective role of copaiba oil–resin against inflammatory bone loss in oral diseases, with the current work providing new insights into collagen preservation in periapical bone.

The current study presents some limitations that should be pointed out. First, the scope was limited to the use of copaiba oil–resin, without combining it with conventional endodontic treatments, which are not intended to be replaced by the proposed therapy. Although reductions in lesion size and improved bone preservation were observed, further research is necessary to evaluate the role of copaiba as an adjuvant to standard treatments. The mechanisms underlying bone preservation remain unclear due to the lack of direct assessment of osteoblast or osteoclast activity. Additionally, the study examined a single species of copaiba from a specific geographic region, which may limit reproducibility. The current study included a qualitative and quantitative analysis of the chemical composition of *Copaifera reticulata* Ducke. For comparative studies and detailed replication, the oil–resin needs to be extracted from trees from the same location. It also needs to be considered that due to biotic and abiotic factors, even if the resin oil is collected from the same tree, variations in its chemical composition may be found (Sousa et al. 2011). Nevertheless, future studies should explore dose–response relationships, long‐term effects, and the integration of copaiba with standard therapies, in order to fully understand its clinical applicability.

Clinically, these findings highlight the potential of copaiba oil–resin as a complementary therapy for apical periodontitis. By reducing bone loss and inflammation while preserving collagen and trabecular structure, copaiba offers a natural alternative for managing alveolar bone damage in inflammatory conditions. This aligns with previous findings on the efficacy of this oil–resin in reducing infectious diseases of the alveolar bone, such as periodontitis (Dos Santos et al. 2022). While promising, further research is essential to establish its role as an adjunct to conventional treatment in clinical settings.

## 5 Conclusions

The current study highlights the remarkable efficacy of copaiba oil–resin in reducing alveolar bone damage and mitigating inflammation in apical periodontitis. The experimental copaiba administration significantly decreased trabecular spacing, indicating enhanced bone density and repair. Systemic administration of copaiba oil–resin presents a promising therapeutic approach that may complement existing treatments for apical periodontitis.

## Author Contributions

D.R.F., R.R.L.: conceptualisation. R.M.F.N., D.R.F., J.N.C., J.M.M.‐S., B.R.R.P.: data curation. R.M.F.N., D.R.F., L.O.B., V.S.C.: formal analysis. R.R.L.: funding acquisition. R.M.F.N., D.R.F., J.D.M.M.: investigation. R.M.F.N., D.R.F., J.N.C., J.D.M.M., R.R.L.: methodology. R.M.F.N., R.R.L.: project administration. R.M.F.N., D.R.F., L.O.B., V.S.C., J.M.M.‐S., B.R.R.P., J.D.M.M.: roles/writing – original draft. R.S.D.C., O.A.L., F.M.C., R.R.L.: writing – review and editing.

## Disclosure

Guidelines: We followed the Guide for the Care and Use of Laboratory Animals (National Research Council, 2011) and adhered to the ARRIVE 2.0 guidelines (Du Sert et al. 2020). Additionally, we used the PRIASE 2021 checklist, a reporting guideline for animal studies in Endodontology (Nagendrababu et al. 2021).

## Ethics Statement

The Ethics Committee on Experimental Animals of the Federal University of Pará (CEUA) approved all procedures (approval number 5496221220).

## Conflicts of Interest

The authors declare no conflicts of interest.

## Supporting information


**Data S1:** iej70063‐sup‐0001‐Supplementary file 1.docx.

## Data Availability

The data that support the findings of this study are available from the corresponding author upon reasonable request.
